# *Desulfovibrio vulgaris* as a model microbe for the study of corrosion under sulfate‐reducing conditions

**DOI:** 10.1002/mlf2.12018

**Published:** 2022-03-24

**Authors:** Toshiyuki Ueki, Derek R. Lovley

**Affiliations:** ^1^ Key Laboratory for Anisotropy and Texture of Materials, Ministry of Education, Electrobiomaterials Institute Northeastern University Shenyang China; ^2^ Department of Microbiology University of Massachusetts Amherst MA USA

**Keywords:** corrosion, *Desulfovibrio*, electron shuttle, extracellular electron transfer, Fe^0^ oxidation, hydrogenase, hydrogen uptake, sulfate reduction, sulfide

## Abstract

Corrosion of iron‐containing metals under sulfate‐reducing conditions is an economically important problem. Microbial strains now known as *Desulfovibrio vulgaris* served as the model microbes in many of the foundational studies that developed existing models for the corrosion of iron‐containing metals under sulfate‐reducing conditions. Proposed mechanisms for corrosion by *D. vulgaris* include: (1) H_2_ consumption to accelerate the oxidation of Fe^0^ coupled to the reduction of protons to H_2_; (2) production of sulfide that combines with ferrous iron to form iron sulfide coatings that promote H_2_ production; (3) moribund cells release hydrogenases that catalyze Fe^0^ oxidation with the production of H_2_; (4) direct electron transfer from Fe^0^ to cells; and (5) flavins serving as an electron shuttle for electron transfer between Fe^0^ and cells. The demonstrated possibility of conducting transcriptomic and proteomic analysis of cells growing on metal surfaces suggests that similar studies on *D. vulgaris* corrosion biofilms can aid in identifying proteins that play an important role in corrosion. Tools for making targeted gene deletions in *D. vulgaris* are available for functional genetic studies. These approaches, coupled with instrumentation for the detection of low concentrations of H_2_, and proven techniques for evaluating putative electron shuttle function, are expected to make it possible to determine which of the proposed mechanisms for *D. vulgaris* corrosion are most important.

## INTRODUCTION

Understanding the mechanisms for the corrosion of metals is key to developing strategies for preventing this economically significant problem^1^, ^2^. Following the first suggestion that microbes might be important catalysts for the corrosion of metals^3^, ^4^, a thorough analysis of the available data led to the conclusion that sulfate‐reducing microorganisms play a key role in iron corrosion^5^. However, in the 1930s, *Spirillium desulfuricans* was the only microbe known to be capable of sulfate reduction^5^. The genus and species names of this and similar strains of sulfate‐reducing bacteria investigated in corrosion studies have changed over time^6^, ^7^, but most are now generally recognized as strains of *Desulfovibrio vulgaris* (Table 1).

**Table 1:** Iron corrosion studies with *Desulfovibrio vulgaris*.

Year	Strain^a^	Iron source	Lactate	Mechanism^b^	Reference
1934	*Spirillum desulfuricans*	CI	+	Ha	^5^
1939	*Vibrio desulfuricans*	MS	+	N	^8^
1947	*Vibrio desulfuricans*	MS	−	N	^9^
1951	*Vibrio desulfuricans*	Armco ingot iron	+ −	Ha	^10^
1952	*Vibrio desulfuricans*	Armco ingot iron	+ −	S	^11^
1960	*D. desulphuricans* Hildenborough NCIB 8303	MS	+	Ha	^12^
1964	*D. desulphuricans* Benghazi NCIB 8401	MS	+?	Ha	^13^
	America NCIB 8372				
	Teddington R NCIB 8312				
	Hildenborough NCIB 8303				
	Llanelly NCIB 8446				
1968	*D. desulfuricans* Teddington R	MS	+	S	^14^
1968	Hildenborough NICB 8303	MS	+	Ha	^15^
1971	*D. desulfuricans* Teddington R NCIB 8312	MS	+ − (+F)	Hs	^16^
1974	Hildenborough NCIB 8303	MS	+	S	^17^
1982	Isolated from River Thames' sediment	MS (EN2)	+	N	^18^
1986	Hildenborough NCIB 8303	MS	− (+A)	Ha	^19^
1986	Marburg DSM 2119 (Postgate and Campbell)	Steel	+ − (+A)	Ha	^20^
1986	Madison	MS	− (+F)	Ha	^21^
1990	Hildenborough NCIB 8303	MS	+	Ha	^22^
1991	DSM 1744 (Postgate and Campbell)	SS (AISI 3161)	−	N	^23^
1991	Not specified	Iron (99%) SS (Fe–15Cr–10Ni)	+	Hs	^24^
1991	Not specified	SS (410)	+	S	^25^
1991	Isolated from cutting oil emulsions	CS (SAE 1020)	+	N	^26^
1992	Woolwich NCIMB 8457	MS (BS970)	+	N	^27^
1993	NCIB 8303	Iron	+	N	^28^
	(Postgate and Campbell)	CS (SAE 1090)			
	(Hildenborough)	SS (18‐8)			
	(DSM 644)				
1994	Not specified	CS (X52)	−	N	^29^
1995	Isolated from cutting oil emulsions	MS	+	N	^30^
1995	Not specified	SS (304)	+	N	^31^
1995	ATCC 25979	SS (304)	+	N	^32^
1997	Not specified	SS (AISI 304L)	+	N	^33^
1997	Not specified	SS (316L)	+	N	^34^
1999	ATCC 29579	MS (SAE 1018)	+	N	^35^
	(Postgate and Campbell)	SS 304			
	(Hildenborough)				
	(NCIB 8303, DSM 644)				
2002	LMG 7563	MS	+	N	^36^
2004	Not specified	Iron	− (+A)	D	^37^
2004	ATCC 29579	MS (1010)	+	N	^38^
2007	DSM 664	MS (BST 503‐2)	+	N	^39^
2008	Hildenborough NCIMB 8303	CS (ASTM A366)	+ − (+A)	Ha	^40^
2008	Isolated from an oil field separator	MS (AISI 1018)	−	N	^41^
2008	DSMZ 644	Iron	+	N	^42^
		CS (ST 37)			
		SS (304)			
2010	DSMZ 644	Alloyed steel (1.4301, UNS 304)	+	N	^43^
2013	ATCC 7757	CS (C1018)	+	N	^44^
	(Postgate and Campbell)				
	(C‐6, CT1, IFO 13699, NCIB 8372)				
2014	ATCC 7757	CS (C1018)	+	N	^45^
2014	ATCC 7757	CS (X70)	+	N	^46^
2014	ATCC 7757	CS (API5L X‐70)	+	N	^47^
2014	ATCC 7757	CS (API5L‐X70)	+	N	^48^
2015	ATCC 7757	CS (C1018)	+	F	^49^
2015	ATCC 7757	SS (304)	+	F	^50^
2016	ATCC 7757	CS (C1018)	+	N	^51^
2016	ATCC 7757	CS (UNS G10100)	+	N	^52^
2016	Hildenborough DSM 644	MS (BST 503‐2)	+	N	^53^
2017	Not specified	CI	+	N	^54^
2017	ATCC 7757	MS	+	N	^55^
2017	ATCC 7757	CS (C1018)	+	N	^56^
2018	ATCC 7757	CS (C1018)	+	S	^57^
2019	Not specified	CS (1018)	+	N	^58^
2019	ATCC 7757	CS (1018)	+	N	^59^
2019	ATCC 7757	PS (X80)	+	N	^59^
2020	Hildenborough	CS (1030)	+	N	^60^
2020	ATCC 7757	SS (2205)	+	N	^61^
2020	Hildenborough	CS	+	N	^62^
2020	ATCC 7757	CS (X65)	+	F	^63^
2021	ATCC 7757	GS	+	N	^64^
2021	ATCC 7757	SS (410, 420, 316, 2206)	+	N	^65^
2021	ATCC 7757	CS (C1018)	+	N	^66^
2021	ATCC 7757	SS (2205)	+	N	^67^
2021	ATCC 7757	SS (2205)	+	N	^68^

^a^ Strains now considered to be *Desulfovibrio vulgaris* were previously designated as *Spirillium desulfuricans*, *Vibrio desulfuricans*, *Desulfovibrio desulfuricans*, and *Desulfovibrio desulphuricans* ^6^, ^7^. Therefore, microbes that were later renamed *D. vulgaris* are listed by the name designated in the original text. Only strain designations are listed for strains designated as *D. vulgaris* in the original text. Alternative designations for these strains are described in parentheses.

^b^ Primary corrosion mechanism discussed. Ha, abiotic H_2_ production from iron; Hs, H_2_ production from iron‐catalyzed by FeS mineral deposits; S, sulfide promoting iron corrosion; D, direct electron transfer; F, electron transfer with a flavin shuttle; N, not applicable (the studies focused on biofilm formation, growth inhibition, corrosion inhibition, etc.). +, lactate included; −, no lactate; − (+A), no lactate but acetate added; − (+F), cells grown on fumarate; CI, cast iron; CS, carbon steel; GS, galvanized steel; MS, mild steel; PP, pipeline steel; SS, stainless steel.

There is substantial evidence that *Desulfovibrio* species are involved in the corrosion of iron‐containing metals in anaerobic environments^69^, ^70^. *Desulfovibrio* species were abundant within the microbial community on metal surfaces exposed to oil field production waters^71^, ^72^, corroded oil pipelines^73^, corroding steel pipe carrying oily seawater^74^, rust layers on steel plates immersed in seawater^75^, and the inner rust layer on carbon steel^76^. *Desulfovibrio* species were recovered in culture from corrosion sites^77^, ^78^, ^79^, ^80^, including a *D. vulgaris* strain isolated from an oil field separator in the Gulf of Mexico that was damaged by corrosion^41^. Microbial activity on the cathodes of bioelectrochemical systems is thought to be related to microbial corrosion^81^ and *Desulfovibrio* species are often enriched on cathodes from diverse microbial communities^82^, ^83^, ^84^.

**Figure 1:**
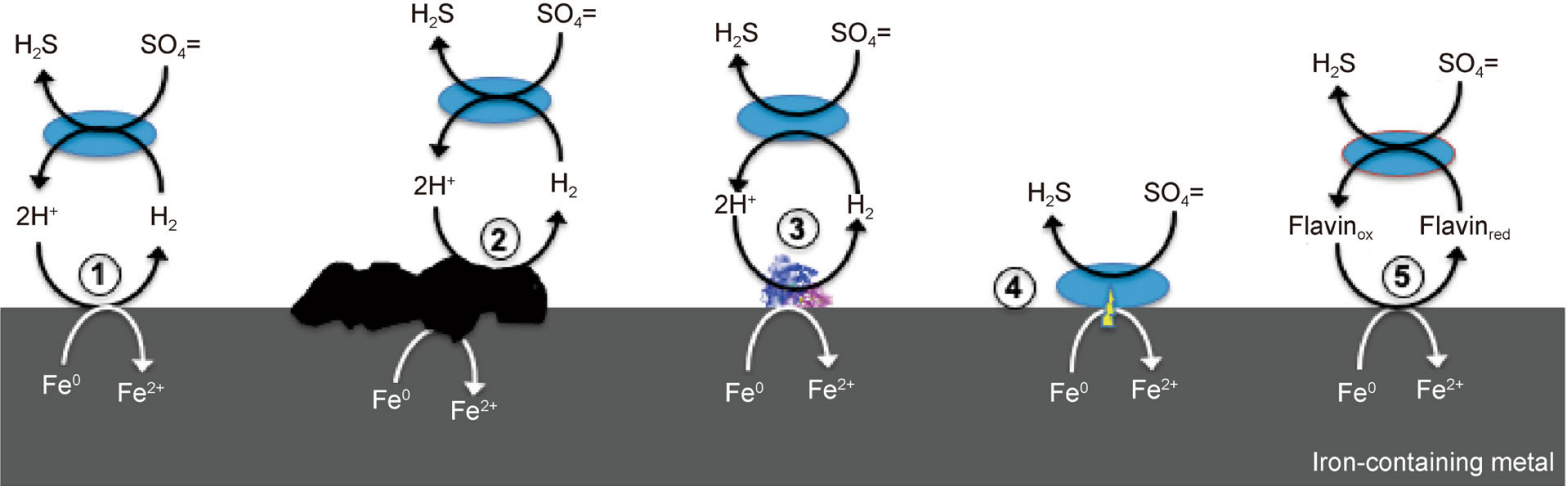
Previously proposed mechanisms for *Desulfovibrio vulgaris* to promote the corrosion of iron‐containing metals. These include the consumption of abiotically produced H_2_ (1); consumption of H_2_ generated via catalysis by FeS (2) or hydrogenase (3); direct electron transfer from Fe^0^ to cells via outer‐surface electron transport components on the cell surface (4); and Fe^0^ oxidation via reduction of the oxidized form of soluble flavin electron shuttle (Flavin_ox_) with reduced flavin (Flavin_red_) serving as the electron donor for sulfate reduction (5). The studies proposing these mechanisms are cited in the main text.

Several mechanisms for *D. vulgaris* corrosion of iron‐containing metals have been proposed (Figure 1). These mechanisms may not be mutually exclusive. As detailed in this review, each of these models still requires rigorous examination. However, with the increasing availability of molecular tools to probe microbial activity and tools for genetic manipulation of *D. vulgaris* ^85^, ^86^, it now may be the time to either eliminate or confirm some of the existing mechanistic models for *D. vulgaris* corrosion or to develop new models. The purpose of this review is to summarize the previously proposed routes for *Desulfovibrio* species iron corrosion and to suggest experimental approaches to further advance the understanding of corrosion by this popular model microbe.

## IRON CORROSION VIA AN H_2_ INTERMEDIATE

The corrosion of iron‐containing metals results from the oxidation of metallic iron to ferrous iron:

(1) $${\text{Fe}}^{0}\to {\text{Fe}}^{2+}+2e^{-}.$$

As recognized in the early analysis of corrosion by sulfate reducers^5^, protons are a likely electron acceptor for the electrons derived from Fe^0^. In early studies, the product of proton reduction is often referred to as “metallic hydrogen,” but in the absence of data demonstrating that this form of hydrogen exists on the surface of corroding iron or can serve as an electron donor for microbial respiration, we assume that proton reduction yields H_2_, a known electron donor for diverse microbes:

(2) $${\text{Fe}}^{0}+2H^{+}\to {\text{Fe}}^{2+}+H_{2}.$$

H_2_ is an electron donor for *D. vulgaris*:

(3) $$4H_{2}+{{\text{SO}}_{4}}^{2-}\to S^{2-}+4H_{2}O,$$

and growth on H_2_ is possible if acetate is provided as a carbon source^87^.

Substantial abiotic H_2_ production from Fe^0^ was discounted in the early version of the model in which H_2_ serves as an electron carrier between Fe^0^ and cells^5^. However, the mechanism by which cells promoted the oxidation of Fe^0^ with the production of H_2_ was not specified. It is now known that the extent of abiotic H_2_ production depends upon the form of the iron‐containing metal. For example, pure Fe^0^ abiotically produces substantial H_2_ when submerged in anoxic water at circumneutral pH whereas 316 stainless steel does not^88^, ^89^.

This difference in H_2_ production between Fe^0^ and stainless steel could provide one method for evaluating whether *D. vulgaris* relies on H_2_ production to consume electrons from iron‐containing metals. The closely related sulfate reducer *D. ferrophilus* reduced sulfate when pure Fe^0^ was the electron donor, but not in a medium with stainless steel^90^. In contrast, *Geobacter* species capable of direct electron uptake could use either iron form as an electron donor^88^, ^89^, ^90^. These results indicated that *D. ferrophilus* was incapable of direct electron uptake and required the production of H_2_ to mediate electron transfer between Fe^0^ and cells.

The most direct approach to evaluating whether H_2_ is an important intermediate in electron uptake from extracellular electron donors may be to generate a mutant that is unable to consume H_2_ ^91^. *D. vulgaris* Hildenborough has multiple hydrogenases that have different localizations and metal constituents: the periplasmic [NiFe] HynAB‐1 and HynAB‐2, the periplasmic [Fe] HydAB, the periplasmic [NiFeSe] HysAB, the cytoplasmic [Fe] HydC, and the cytoplasmic membrane‐bound Coo and Ech hydrogenases^92^. Deletions of genes for HydAB, HynAB‐1, or HysAB negatively impacted the growth of *D. vulgaris* Hildenborough with H_2_ as the electron donor^93^, ^94^, ^95^. However, these single‐gene deletion mutants and a double deletion mutant of HynAB‐1 and HydAB^95^ still grew on H_2_ as the electron donor, indicating redundant, complementary functions of the multiple hydrogenases. Thus, the construction of a strain with multiple hydrogenase gene deletions may be required to rigorously evaluate the role of H_2_ in corrosion.

Transcriptomic analysis comparing growth on H_2_ supplied from proton reduction with an iron electrode poised at −1.1 V versus growth on H_2_ simply bubbled into medium revealed that the genes for HynAB‐1 and HydAB were more highly expressed during growth on the cathodic H_2_ ^40^. Gene transcripts for HysAB were more abundant when H_2_ was bubbled into the medium. Gene deletions that prevented the function of the HynAB‐1 and HydAB hydrogenases inhibited electron uptake from the iron cathodes^40^, as might be expected for cathodes poised at a low potential to induce H_2_ production. The impact of the hydrogenase gene deletions on corrosion of iron that was not artificially poised at a negative potential was not determined because wild‐type cells could not be grown under these conditions^40^. Lack of growth on unpoised iron suggests an inability to use Fe^0^ as an electron donor. This difference between artificially poised iron cathodes and unpoised iron metal is an important consideration when evaluating other studies^19^ that have concluded that H_2_ is an important intermediate in iron corrosion by *D. vulgaris* based on experiments with electrochemically poised iron electrodes.

However, there is some indirect evidence for H_2_ serving as an intermediary electron carrier between Fe^0^ and *D. vulgaris*, especially when H_2_ is not the sole electron donor. *D. vulgaris* did not reduce sulfate when steel wool was provided as the sole electron donor, but when lactate was added as an additional electron donor, more sulfide was produced than was possible from lactate oxidation alone^20^. In contrast, *D. sapovorans*, which cannot utilize H_2_, did not produce substantially more sulfide when grown in the presence of lactate and steel wool, than when grown with lactate alone. These results suggested that H_2_ was an intermediary electron carrier for *D. vulgaris* electron uptake from the steel wool during growth with lactate^20^. The expression of one or more uptake hydrogenases is expected to be upregulated when H_2_ is serving as an electron donor^96^, ^97^, ^98^. Thus, transcriptional and/or proteomic studies may be useful in further assessing the role of H_2_ as an electron donor during corrosion in the presence of lactate. Additional indirect evidence for the importance of H_2_ as an electron carrier was the finding that *D. vulgaris* corroded “mild steel” faster than the gram‐positive *D*. *orientis*, which cannot consume H_2_ ^12^, ^13^.

Other early studies suggested that H_2_ production from iron was not a mechanism for corrosion^10^, ^11^. However, the medium for investigating the possibility for H_2_ serving as an electron donor did not include acetate, which is required as a carbon source for growth on H_2_. Therefore, no conclusion on the role of H_2_ is possible from those studies.

## FACTORS PROMOTING H_2_ PRODUCTION

In a diversity of microbes, hydrogenases released from lysed cells, or specifically transported to the outer surface of living cells, facilitate the production of H_2_ from Fe^0^ ^99^, ^100^, ^101^, ^102^. For example, methanogens highly effective in corrosion can produce an extracellular hydrogenase that enhances H_2_ production from Fe^0^ ^99^. Such extracellular hydrogenases have not been reported in *Desulfovibrio* species, but moribund cells of *D. vulgaris* release periplasmic hydrogenases that can retain activity for months^103^. Subjecting *D. vulgaris* to starvation, a condition likely to promote cell death and lysis, enhanced corrosion^45^, ^58^. Therefore, studies to evaluate the role of extracellular hydrogenases in corrosion by *D. vulgaris* are warranted.

The iron sulfide that precipitates on iron‐containing metals during corrosion coupled to sulfate reduction may also increase H_2_ production. The addition of FeS reduced the overpotential necessary to produce H_2_ from iron cathodes, suggesting a role for FeS in promoting the formation of H_2_ ^16^. In studies in which the culture was grown on fumarate rather than via sulfate reduction, the current was generated at more positive potentials when FeS was deposited on either mild steel or platinum cathodes^14^. These results further indicate that FeS may serve as a catalyst for H_2_ generation. However, in studies with carbon steel coupons, it appeared that higher accumulations of sulfide inhibited corrosion^57^. The ability of *D. vulgaris* to corrode steel with either benzyl viologen as the electron acceptor^15^ or when growing on fumarate^21^ demonstrated that sulfide production was not essential for corrosion. Thus, a clear‐cut concept for the role of FeS in corrosion has yet to be established.

Technology for measuring H_2_ concentrations at extremely low concentrations during corrosion is available^89^. Thus, with the appropriate H_2_ detector it should be possible to directly evaluate the role of FeS in facilitating H_2_ production from iron‐containing metals, simply by monitoring H_2_ generation in the presence or absence of different quantities of FeS precipitate.

## ELECTRON SHUTTLES OTHER THAN H_2_

Soluble redox‐active molecules promote extracellular electron exchange between microbes and minerals, electrodes, and other microbial species^104^, ^105^, ^106^, ^107^. These electron shuttles typically accelerate extracellular electron exchange by alleviating the need for outer‐surface electron transfer components to establish direct electrical contact with particulate extracellular donors and acceptors. The addition of riboflavin and flavin adenine dinucleotide enhanced *D. vulgaris* corrosion of carbon steel and stainless steel^49^, ^50^, ^63^. However, amendments of these cofactors, which are important for the function of numerous proteins, could influence *D. vulgaris* growth and metabolism in many ways. To determine whether flavins can serve as an electron shuttle for the corrosion of iron‐containing metals coupled to sulfate reduction it is necessary to demonstrate that: (1) the metals are capable of reducing the flavins; and (2) that the reduced flavins can serve as electron donors for sulfate reduction.

## DIRECT ELECTRON UPTAKE

Direct electron uptake from iron‐containing metals has been demonstrated with *Geobacter sulfurreducens* and *Geobacter metallireducens* ^88^, ^89^, ^90^. Strains unable to utilize H_2_ readily reduced fumarate, nitrate, or Fe(III) with pure Fe^0^ or stainless steel as the electron donor. Deletion of genes for outer‐surface, multiheme *c*‐type cytochromes previously shown to be involved in electron exchange with other extracellular donors/acceptors inhibited the corrosion.

There are no examples of similar studies with sulfate‐reducing microorganisms. It was suggested that *c*‐type cytochromes positioned in the outer membrane of *D. vulgaris* might be able to make an electrical connection with Fe^0^ ^108^. However, subsequent studies have indicated that *D. vulgaris* does not have outer‐surface cytochromes^92^. Clear next steps in this line of investigation would be to rigorously verify whether cytochromes are exposed on the outer surface of *D. vulgaris*, and if so, evaluate their role in iron corrosion with the appropriate gene deletion studies. Genetic, biochemical, biophysical, and immunological approaches previously employed for investigating the location and function of the outer‐surface cytochromes of *Geobacter* would be suitable^109^, ^110^, ^111^, ^112^.

Although it has been suggested that several of the *c*‐type cytochromes of *D. ferrophilus* may be localized in the outer membrane, no genetic studies have been conducted to determine whether they are involved in extracellular electron exchange^90^, ^113^, ^114^, ^115^. As noted above, experimental analysis of iron corrosion by *D. ferrophilus* has suggested that it relies on H_2_ as an intermediary electron carrier rather than direct electron uptake^90^.

## CONCLUSIONS AND FUTURE DIRECTIONS

Sulfate‐reducing microorganisms are considered to be important agents for catalyzing the corrosion of iron‐containing metals and *D. vulgaris* has historically been the model microbe of choice for elucidating the mechanisms for corrosion by sulfate reducers. As summarized above (Figure 1), previous studies have suggested several mechanisms by which *D. vulgaris* may enhance corrosion, but each of the proposed mechanisms requires further experimental evaluation. The mechanisms for electron transfer between iron‐containing metals and *Geobacter* species were elucidated with genome‐scale transcriptomics coupled with phenotypic analysis of mutant strains in which the genes for proteins hypothesized to be involved in electron transfer were deleted^88^, ^89^. Deletion of the genes for hydrogenases and hypothesized outer‐surface electrical contacts made it possible to determine the role of H_2_ as an intermediary electron carrier and to identify likely electrical contacts on the outer surface of the cell. A similar approach seems possible for the study of *D. vulgaris* corrosion mechanisms. Transcriptomics of *D. vulgaris* biofilms is possible^39^, ^40^ to aid in identifying components that may have increased expression during corrosion of iron‐containing metals versus other growth modes. Other candidates for corrosion components may be identified from the known physiological roles of proteins or their cellular location. Methods for the targeted deletion of genes in *D. vulgaris* are available^85^, ^86^ making it possible to evaluate the function of proteins hypothesized to be of importance. In fact, an extensive library of *D. vulgaris* mutants is publicly available, potentially eliminating the substantial investment of time and resources required for mutant construction^86^. Comparison of corrosion capabilities with Fe^0^, which readily reduces protons to generate H_2_, versus stainless steel, which does not generate H_2_, provides an additional tool to evaluate the role of H_2_ as an intermediary electron carrier for corrosion^89^, ^90^. Mechanisms for the corrosion of carbon steel, the most common form of iron in structural materials, should also be investigated. The hypothesis that FeS deposits stimulate H_2_ production from iron‐containing metals should be readily addressable with highly sensitive H_2_ detection systems^88^, ^89^. Mechanistic and genetic^116^, ^117^, ^118^, ^119^, ^120^ approaches for the study of the role of electron shuttles in electron transfer to minerals and electrodes should be applicable to the study of the role of flavins as electron shuttles for corrosion. Therefore, it is expected that *D. vulgaris* will continue to serve as an important model microbe for the further elucidation of mechanisms for corrosion under sulfate‐reducing conditions.
